# Monitoring the goals of the plans for coping with Chronic Non-Communicable Diseases: results of the National Health Survey, Brazil, 2013 and 2019

**DOI:** 10.1590/SS2237-9622202200008.especial

**Published:** 2022-07-06

**Authors:** Deborah Carvalho Malta, Alanna Gomes da Silva, Crizian Saar Gomes, Sheila Rizzato Stopa, Max Moura de Oliveira, Luciana Monteiro Vasconcelos Sardinha, Roberta Betânia Caixeta, Cimar Azeredo Pereira, Eduardo Luiz Gonçalves Rios-Neto

**Affiliations:** 1 Universidade Federal de Minas Gerais, Escola de Enfermagem, Belo Horizonte, MG, Brazil; 2 Universidade Federal de Minas Gerais, Programa de Pós-Graduação em Enfermagem, Belo Horizonte, MG, Brazil; 3 Universidade Federal de Minas Gerais, Programa de Pós-Graduação em Saúde Pública, Belo Horizonte, MG, Brazil; 4 Ministério da Saúde, Departamento de Análise de Situação de Saúde, Brasília, DF, Brazil; 5 Universidade Federal de Goiás, Instituto de Patologia Tropical e Saúde Pública, Goiânia, GO, Brazil; 6 Pan American Health Organization, Washington, DC, United States; 7 Instituto Brasileiro de Geografia e Estatística, Rio de Janeiro, RJ, Brazil

**Keywords:** Chronic Disease, Health Surveys, Goals, Brazil

## Abstract

**Objective:**

To monitor the achievement of the action plans for the prevention and control of Non-Communicable Diseases agreed-upon targets.

**Methods:**

Cross-sectional study, with data from the 2013 and 2019 National Health Survey. The following targets, up to 2025, were evaluated: physical inactivity, alcohol consumption, salt/sodium, tobacco use, high blood pressure, diabetes, overweight, obesity, cervical cytology testing, and drug therapy and counseling. To check whether the targets were achieved, the prevalence ratio was calculated (PR).

**Results:**

60,202 individuals were assessed in 2013, and 88,531 in 2019. The targets for physical inactivity (PR = 0.88; 95%CI 0.86;0.90) and cervical cytology coverage (79.4%; 95%CI 78.3;80.3) were achieved. Tobacco use was reduced, albeit below the target. The prevalence of hypertension, diabetes, overweight, obesity and alcohol consumption increased, and the targets will not be attained.

**Conclusion:**

Two indicators reached the agreed targets, however it is necessary to advance in actions and policies to meet the others.

**Study contributions**

**Main results**

The targets for physical inactivity and Pap smear coverage have been attained. Tobacco use has been reduced, although below the target. The prevalence of hypertension, diabetes, overweight, obesity and use of alcohol have increased and the targets will not be met.

**Implications for services**

Monitoring the agreed upon targets for tackling the non-communicable diseases (NCDs) can provide support in planning, control, prevention and treatment actions concerning such diseases and also the evaluation processes.

**Perspectives**

There still remains the need for effective policies for the control and prevention of NCDs, advancement in legal and regulatory measures, as well as intersectoral articulations, and reducing the socioeconomic and health inequalities.

## Introduction

Globally, Non-Communicable Diseases (NCDs) are the main cause of death and represent a threat to all nations.^1^ They financially impact individuals, families, communities and governments, due to premature deaths, disability, treatments and hospitalizations.^2^

Annually, NCDs are responsible for over 70% of all deaths worldwide (41 million), of which 15 million are premature deaths (people between the ages of 30 and 69).^1^ The burden of NCDs is higher in low- and middle-income countries, accounting for approximately 78% of the total mortality.^1^ In Brazil, in 2019, overall mortality due to these diseases corresponded to about 74% of all deaths (975,400 deaths)^1^ and 71% of the total disability-adjusted life years (DALYs).^3^ Premature deaths, in 2017, corresponded to 41.3% (556,639).^4^

These diseases are multifactorial, however, the main causes include modifiable risk factors (RFs), such as tobacco use, harmful use of alcohol, physical inactivity and unhealthy diets. Adding to these, there are underlying social determinants that impact the increase and severity of NCDs and their RFs.^5^ ^,^ ^6^

Several national and international initiatives have been promoted, in an effort to reduce the impact of NCDs. In September 2011, the High Level Meeting on Prevention and Control of Non-communicable Diseases of the United Nations (UN) was held. Its aim was to discuss the global commitments in terms of NCDs, which resulted in a political declaration in which all member states made a commitment to detain the increase of these diseases, by means of preventive actions regarding their main RFs and ensuring adequate attention to healthcare.^6^ It is worth highlighting that, in that meeting, was presented the 2011-2022 Strategic Action Plan to Tackle Non-communicable Diseases (NCDs) in Brazil.^6^ In 2013, the World Health Organization (WHO) approved the Global Action Plan for the Prevention and Control of NCDs, which contains the targets for reducing these diseases and their RFs by 2025, as well as the monitoring framework to track their implementation.^7^ In 2015, the UN also included strategies and targets to reduce the prevalence and RFs for NCDs, which will be monitored until 2030.^8^ In the Americas, the 2014-2019 Strategic Plan of the Pan American Health Organization (PAHO), defined the collective priorities and specified the results to be achieved during the specified period.^9^ Box 1 presents the targets for tackling the NCDs, according to the four plans cited above.

**Box 1:** Indicators and respective targets proposed in the Brazilian Strategic Action Plan to Tackle Non-communicable diseases (NCDs); World Health Organization (WHO); Global Action Plan for the Prevention and Control of NCDs; Sustainable Development Goals (SDGs); and the Strategic Plan of the Pan American Health Organization (PAHO)

Indicators	Targets of the Brazilian National Plan (2011-2022)	Targets of WHO Global Plan (2013-2025)	Targets of the SDGs (2015-2030)	Targets of the PAHO Plan (2014-2019)
Premature mortality due to NCDs	Reduce the rate of premature mortality (30 to 69 years of age) due to NCDs by 2% per year.	Reduce premature mortality from cardiovascular disease, cancer, diabetes or chronic respiratory diseases by 25%.	Reduce premature mortality from NCDs by one third through prevention and treatment, and promote mental health and wellbeing.	a
Consumption of alcoholic beverages	Reduce the prevalence of harmful use of alcohol by 10%.	Reduce the harmful use of alcohol, as appropriate, within the national context by at least 10%.	Strengthen the prevention and treatment of substance abuse, including narcotic drug abuse and harmful use of alcohol.	Reduce harmful use of alcohol by 5%, as appropriate within the national context.
Physical activity	Increase the prevalence of leisure time physical activity by 10%.	Reduce the prevalence of insufficient physical activity by 10%.	a	Reduce the prevalence of insufficient physical activity in adults by 5%.
Tobacco use	Reduce the prevalence of smoking in adults by 30%.	Reduce the prevalence of current tobacco use in persons aged 15 or over by 30%.	Strengthen the implementation of the WHO^c^ Framework Convention on Tobacco Control in all countries, as appropriate.	Reduce the prevalence of current tobacco use in individuals aged 15 or over by 30%.
Consumption of fruits and vegetables	Increase the consumption of fruits and vegetables by 10%.	a	a	a
Salt/sodium intake	Reduce the mean consumption of salt/sodium to 5g.	Reduce mean population intake of salt/sodium by 30%.	a	Reduce mean population intake by 30%.
Blood pressure (hypertension)	a	Reduce the prevalence of raised blood pressure or contain the prevalence of raised blood pressure, according to national circumstances, by 25%.	a	Increase the percentage of controlled hypertension at population level (< 140/90 mmHg) among persons 18+ years of age to 35%.
Diabetes	a	Halt the rise in diabetes.	a	Halt the rise in diabetes.
Obesity	Halt the rise of diabetes in adults.	Halt the rise in obesity.	a	Halt the rise in obesity.
Drug therapy to prevent heart attacks and strokes	a	Ensure that at least 50% of eligible people receive drug therapy and counseling (including glycaemic control) to prevent heart attacks and strokes.	a	a
Essential NCDs basic technologies and medicines	a	Increase, to 80%, the availability of the affordable basic technologies and essential medicines, including generics, required to treat major NCDs in both public and private facilities.	Support the research and development of vaccines and medicines for the communicable and non-communicable diseases that primarily affect developing countries, provide access to affordable essential medicines and, in particular, provide access to medicines for all.	a
Mammogram	Increase the coverage of mammograms in women aged 50 to 69 years to 70%.	a	a	a
Preventive cervical cancer screening	Increase the coverage of preventive cervical cancer in women aged 25 to 64 years to 85%.	a	a	

a) Indicators and targets not included in the plans.

Brazil implemented an NCDs Surveillance system, by implementing different population surveys, such as the National Health Survey (*Pesquisa Nacional de Saúde* - PNS), providing means to ascertain the burden of these diseases, as well as their RFs and protection burden, in order to support planning, control, prevention and treatment actions, along with evaluation processes.^10^ In this sense, it is essential to monitor the agreed-upon targets for coping with NCDs and their RFs and, thus, to assess their reach and advancement, in addition to revising strategies when needed.

The aim of this study was to monitor the achievement of the action plans for the prevention and control of Non-Communicable Diseases agreed-upon targets.

## Methods

### Study design

This was a cross-sectional study that used data from the PNS conducted in 2013 and 2019. Data collection for this research took place from August 2013 to February 2014, and from August 2019 to March 2020, respectively.

### Context

The PNS is a nationwide household health survey, conducted by the Brazilian Institute of Geography and Statistics (IBGE), in partnership with the Ministry of Health. A three-stage cluster sampling design was used: census tracts (primary units), households (secondary units), and adult residents aged 18 years or older (tertiary units). It must be noted, however, that in the 2019 PNS, in the third stage, the selected resident could be 15 years or older.^11^ ^,^ ^12^

To calculate the sample size, mean values, variances and effects of the sampling plan were considered, accepting a non-response rate of 20%. In 2013, the sample was around 80 thousand households and, in 2019, it was 108,525.^11^ ^,^ ^12^ In order to compare the two editions of the PNS, a new calibration of the expansion factors of the 2013 PNS was carried out by IBGE, considering the revision of the population projections for Brazil and Federative Units, by sex and age, and the same population projection was used to calibrate the weights of the 2019 PNS.^13^

The database and questionnaires for the 2013 and 2019 PNS are open access for public use, available from https://www.pns.icict.fiocruz.br/, and was accessed in December 08, 2020.

### Participants

For the present study, data pertaining to the selected residents aged 18 years and older from the two editions of the PNS were used.

### Variables

The indicators evaluated by the 2013 and 2019 PNS, and the respective targets proposed in WHO Global Action Plan for the Prevention and Control of NCDs, were used:^7^

- Harmful use of alcohol: proportion of adults who reported the consumption of five or more drinks in the case of males and four or more drinks, in the case of females, on one occasion in the past 30 days. In 2019, heavy drinking was defined as five or more drinks on one occasion for both sexes, due to changes in the questionnaire. *Target:* to reduce the prevalence of harmful use of alcohol by at least 10%;
- Insufficient physical activity: proportion of adults who did not get at least 150 minutes of physical activity per week, considering leisure time, work and commute. *Target:* to reduce the prevalence of insufficient physical activity by 10%;
- Tobacco use: proportion of current users of tobacco products. *Target:* to reduce the current prevalence of tobacco use by 30%;
- Intake of salt/sodium: mean intake of salt by the Brazilian population, estimated through urinary sodium excretion. Data from the 2014/2015 PNS laboratory tests database were analyzed. *Target:* to reduce the mean population intake of salt/sodium by 30%;
- Raised blood pressure: Proportion of adults with elevated blood pressure (hypertension). Individuals with blood pressure ≥ 140mmHg/≥ 90mmHg were considered hypertensive. For this indicator, data measured by the 2013 PNS, which are available only for that year, were used. As an alternative measure, the self-reported blood pressure was also evaluated, calculated according to the responses to the following question: *Has a doctor ever told you that you have hypertension (high blood pressure)?* (yes; no) Those who answered “yes” were considered hypertensive. *Target:* to reduce the prevalence of high blood pressure or to contain its prevalence, according to national circumstances, by 25%;
- Diabetes: proportion of diabetic adults, considering those with glycated hemoglobin ≥ 6.5% or using medication, by using the 2014/2015 PNS laboratory tests database. As an alternative measure, self-reported diabetes was also evaluated, calculated according to the responses to the following question: *Has a doctor ever told you that you have diabetes?* (yes; no) *Target:* to halt the rise in diabetes;
- Overweight and obesity: proportion of adults with excessive weight and obesity. Individuals with body mass index (BMI) ≥ 25 kg/m^2^ and ≥ 30 kg/m^2^, respectively, and whose height and weight were measured, were considered. It is worth noting that for 2019 the anthropometric measurements are available only at national level, impeding an evaluation per region. *Target:* to halt the rise in overweight and obesity;
- Drug therapy and counseling (including glycaemic control) to prevent heart attacks and strokes: to ensure that at least 50% of eligible persons, with cardiovascular risk (CVR) > 30%, receive drug therapy and counseling (including glycaemic control) to prevent heart attacks and strokes. This target is only available for 2013, as the biochemical measurements were only conducted at that time. *Target:* to increase, to 50%, the number of eligible people receiving drug therapy and counseling;
- Preventive cervical cancer screening (Pap smear) coverage for women between the ages 30 to 49: having been screened for cervical cancer at least once in a lifetime, or according to the country’s protocols. *Target:* to increase cervical screening coverage to 70% by 2019 in women aged 30 to 49 years.^9^

### Statistical Analyses

Prevalence and 95% confidence intervals (95%CI) of the 2013 and 2019 indicators were calculated for the total population, according to sex (male; female), level of education (no schooling and incomplete primary education; complete primary education and incomplete secondary education; complete secondary education and incomplete higher education; complete higher education) and region (North; Northeast; Southeast; South; Midwest).

To verify whether the targets had been met, data from 2013 and 2019 were compared, considering the prevalence of the indicators in 2013 as the reference point, through the calculation of the prevalence ratio (PR). The PR were estimated using the Poisson regression model with robust variance. To analyze the PNS data, due to the complex sampling design and distinct selection probabilities, the use of expansion factors or sample weighting for the selected households and selected residents was necessary.^11^ ^,^ ^12^

For data analysis, the software for Statistics and Data Science (Stata Corp LP, College Station, Texas, United States), version 14.0, survey module was used.

### Ethical aspects

The 2013 and 2019 PNS were approved by the National Research Ethics Committee of the National Health Council, under Opinion No. 328,159 and 3,529,376, respectively.

## Results

In the 2013 PNS 60,202 adult individuals were assessed, and 88,531 in the 2019 PNS.

**Table 1:** Prevalence of the indicators for monitoring the targets of the plans for tackling Non-Communicable Diseases (NCDs) in the Brazilian adult population, National Health Survey (PNS), 2013 (n = 60,202) and 2019 (n = 88,531)

Indicators/Targets	2013 % (95%CI^b^)	2019 % (95%CI^b^)	PR^c^ (95%CI^b^)	Target
Harmful use of alcohol	13.6 (13.1;14.2)	17.1 (16.6;17.5)	1.25 (1.19;1.31)	Reduce by 10% (*Target will most likely not be attained*)
Insufficient physical activity	45.7 (44.9;46.5)	40.3 (39.6;40.9)	0.8 (0.86;0.90)	Reduce by 10% (*Target partially attained*)
Tobacco use	14.9 (14.4;15.4)	12.8 (12.4;13.2)	0.8 (0.82;0.90)	Reduce by 30% (*Target partially attained*)
Intake of salt/sodium^a^	9.3 (9.27; 9.41)	d	d	Reduce by 30% (*Target will most likely not be attained*)
Measured hypertension	22.8 (22.1;23.4)	d	d	Reduce by 25% (*Target will most likely not be attained*)
Self-reported hypertension	21.4 (20.8;22.0)	23.9 (23.4;24.4)	1.1 (1.08;1.16)	Reduce by 25% (*Target will most likely not be attained*)
Diabetes measured by glycated hemoglobin ≥ 6.5% or use of medication	8.4 (7.6;9.1)	d	d	Halt the rise (*Target will most likely not be attained*)
Self-reported diabetes	6.2 (5.9;6.6)	7.7 (7.4;8.0)	1.2 (1.16;1.33)	Halt the rise (*Target will most likely not be attained*)
Overweight	56.9 (56.1;57.9)	60.3 (57.7;62.8)	1.0 (1.01;1.11)	Halt the rise (*Target will most likely not be attained*)
Obesidade	20.8 (20.2;21.4)	25.9 (22.5;29.6)	1.2 (1.08;1.43)	Halt the rise (*Target will most likely not be attained*)
Drug therapy to prevent heart attacks and strokes	51.2 (49.7;59.9)	d	d	Reach at least 50% of eligible persons^e^ (*Target attained*)
Preventive cervical cancer screening (30 to 49 years of age)	f	f	f	Increase screening coverage to 70% (*Target attained*)

a) Estimate of sodium excretion from urine sample collected in the 2013 PNS; b) 95%CI: 95% confidence intervals; c) PR: Prevalence ratio; d) Data not collected in the 2019 PNS; e) As the indicators were not collected by the PNS 2019, it was not possible to monitor the target; f) Information not available.

Tables 1 and 2 present the prevalence of the indicators in 2013 and 2019 for the total population and according to sex, respectively. Regarding harmful alcohol consumption, the prevalence for the total population was 13.6% (95%CI 13.1;14.2) in 2013 and 17.1% (95%CI 16.6;17.5) in 2019, with an increase of 25% (PR = 1.25; 95%CI 1.19;1.31). Therefore, the 10% reduction target was not achieved.

**Table 2:** Prevalence of the indicators for monitoring the targets of the plans for tackling Non-Communicable Diseases (NCDs) in the Brazilian adult population, by sex, National Health Survey (PNS), 2013 (n = 60,202) and 2019 (n = 88,531)

Indicators/Targets	Male			Female		
	2013% (95%CI^b^)	2019% (95%CI^b^)	PR^c^ (95%CI^b^)	2013% (95%CI^b^)	2019% (95%CI^b^)	PR^c^ (95%CI^b^)
Harmful use of alcohol	21.5 (20.6;22.4)	26.0 (25.2;26.8)	1.2 (1.14;1.27)	6.6 (6.1;7.1)	9.2 (8.7;9.7)	1.3 (1.28;1.52)
Insufficient physical activity	39.3 (38.2;40.4)	32.1 (31.2;32.9)	0.8 (0.78;0.85)	51.3 (50.3;52.4)	47.5 (46.6;48.3)	0.9 (0.90;0.95)
Tobacco use	19.1 (18.3;20.0)	16.2 (15.6;16.9)	0.8 (0.80;0.90)	11.2 (10.6;11.7)	9.8 (9.4;10.3)	0.8 (0.82;0.94)
Intake of salt/sodium^a^	9.6 (9.52; 9.74)	d	d	9.0 (8.99;9.17)	d	d
Measured hypertension	25.8 (24.8;26.7)	d	d	20.0 (19.3;20.8)	d	d
Self-reported hypertension	18.1 (17.3;18.9)	21.1 (20.4;21.8)	1.1 (1.10;1.23)	24.3 (23.5;25.2)	26.4 (25.8;27.2)	1.0 (1.04;1.13)
Diabetes measured by glycated hemoglobin ≥ 6.5% or use of medication	6.9 (5.9;7.9)	d	d	9.7 (8.6;10.7)	d	d
Self-reported diabetes	5.3 (4.8;5.9)	6.9 (6.5;7.4)	1.3 (1.16;1.46)	7.0 (6.6;7.5)	8.4 (8.0;8.9)	1.2 (1.10;1.30)
Overweight	55.5 (54.4;56.6)	57.5 (54.6;60.4)	1.0 (0.98;1.09)	58.2 (57.2;59.2)	62.6 (58.6; 66.5)	1.0 (1.01;1.15)
Obesity	16.8 (15.9;17.7)	21.8 (18.8;25.1)	1.3 (1.11;1.51)	24.4 (23.6;25.3)	29.5 (24.9;34.6)	1.2 (1.02;1.43)
Drug therapy to prevent heart attacks and strokes	54.8 (43.4;59.0)	d	d	58.1 (51.4;64.6)	d	e
Preventive cervical cancer screening (30 to 49 years of age)	e	e	e	79.4 (78.3;80.3)	e	e

a) Estimate of sodium excretion from urine sample collected in the 2013 PNS; b) 95%CI: 95% confidence intervals; c) PR: Prevalence ratio; d) Data not collected in the 2019 PNS; e) Information not available.

Insufficient physical activity reduced 12% (PR = 0.88; 95%CI 0.86;0.90), from 45.7% (95%CI 44.9;46.5) in 2013 to 40.3 % (95%CI 39.6;40.9) in 2019. Hence, the 10% reduction target was attained for the total population. However, when considering sex, male individuals reduced insufficient physical activity by only 10%.

 Tobacco consumption went from 14.9% (95%CI 14.4;15.4) in 2013 to 12.8% (95%CI 12.4;13.2) in 2019, with a reduction of 14% (PR = 0.86; 95%CI 0.82;0.90); therefore, the reduction target of 30% for tobacco use was not achieved.

Mean salt intake, which was 9.3g/day (95%CI 9.27;9.41) in 2013, was not measured in 2019, which made it impossible to monitor the target of reducing salt consumption by 30% until 2025. In addition, the prevalence of measured hypertension was 22.8% (95%CI 22.1;23.4) in 2013 and, as occurred with salt consumption, there was no measurement in 2019. In this case, there are only the self-reported prevalences in 2013 and 2019, which were 21.4% (95%CI 20.8;22.0) and 23.9% (95%CI 23.5;24.4), respectively, representing an increase of 12% (PR = 1.12; 95%CI 1.08;1.16). According to the self-report method, the goal of reducing the prevalence of hypertension by 25% would not be achieved. Likewise, the prevalence of diabetes in 2013, measured by glycated hemoglobin and the use of medications, showed a prevalence of 8.4% (95%CI 7.6;9.1), however, it was not measured in the same way in 2019. When evaluating the self-reported prevalence, there was an increase of 24% (PR = 1.24; 95%CI 1.16;1.33), from 6.2% (95%CI 5.9;6.8) in 2013 to 7.7% (95%CI 7.4;8.0) in 2019. Thus, the aim of halting the rise of diabetes was not attained.

Overweight and obesity increased 6% (56.9% in 2013 and 60.3% in 2019; PR = 1.06; 95%CI 1.01;1.11) and 24% (20.8% in 2013 and 25.9% in 2019; PR = 1.24 95%CI 1.08;1.43), respectively, and therefore it was not possible to achieve the target of these indicators. On the other hand, the target of increasing the number of eligible persons receiving drug therapy and counseling to 50% was attained in 2013, as the prevalence was 51.2%. The target of achieving a 70% coverage for Pap smears in women aged 30 to 49 years, once or more in their lifetime, was also achieved in 2013, with a coverage of 79.4% (95%CI 78.3;80.3%).

**Figure 1:**
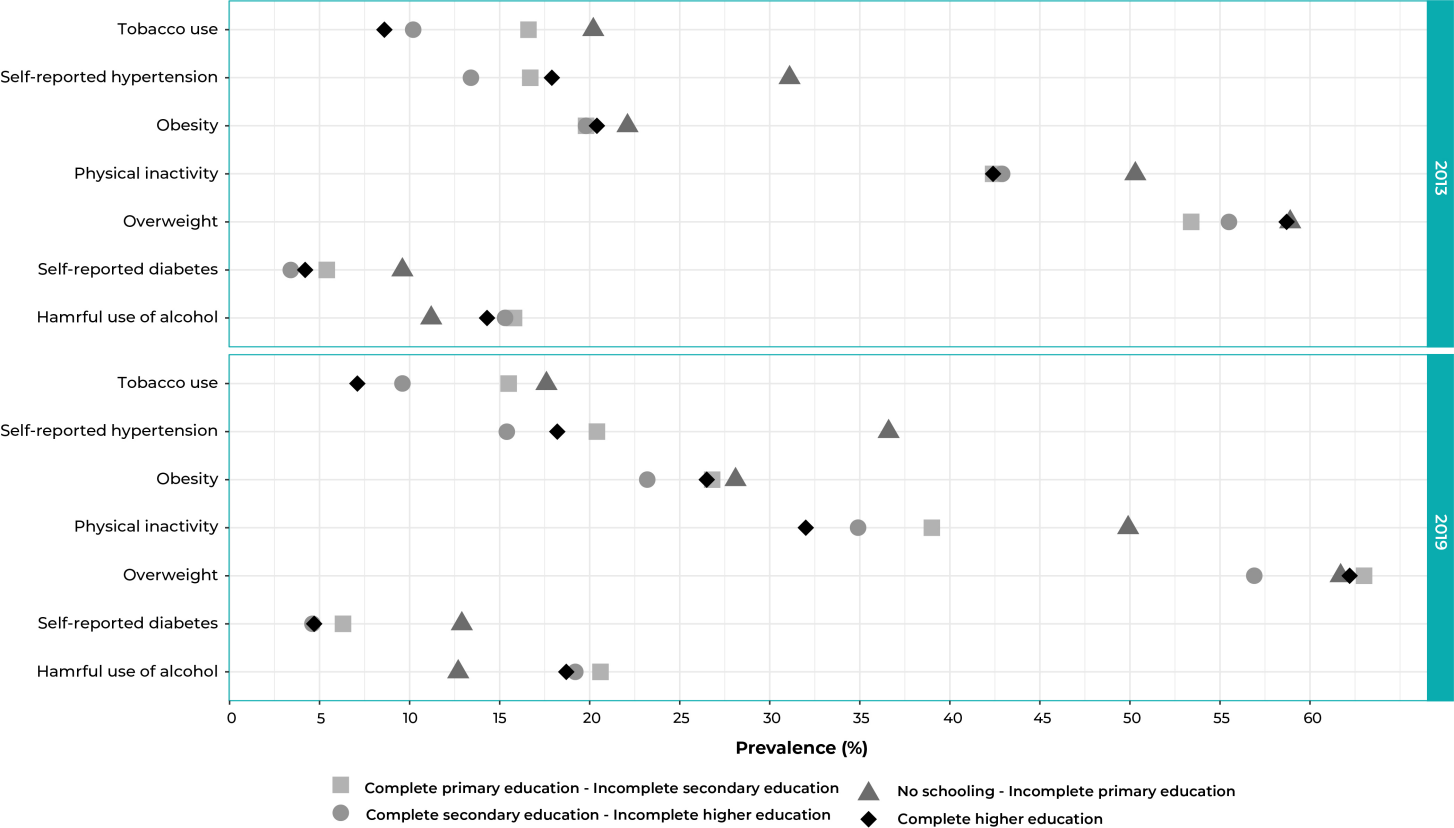
Prevalence of the indicators for monitoring the targets of the plans for tackling Non-Communicable Diseases (NCDs) in the Brazilian adult population, by level of education, National Health Survey (PNS), 2013 (n=60,202) and 2019 (n=88,531)

Figure 1 shows the prevalence for the indicators in 2013 and 2019, according to level of education. The targets for reducing harmful use of alcohol, tobacco use, high blood pressure, diabetes, overweight and obesity were not attained in any stratum. Moreover, the goal of reducing physical inactivity was not achieved in the population with no schooling/incomplete primary education and complete primary education/incomplete secondary education.

**Figure 2:**
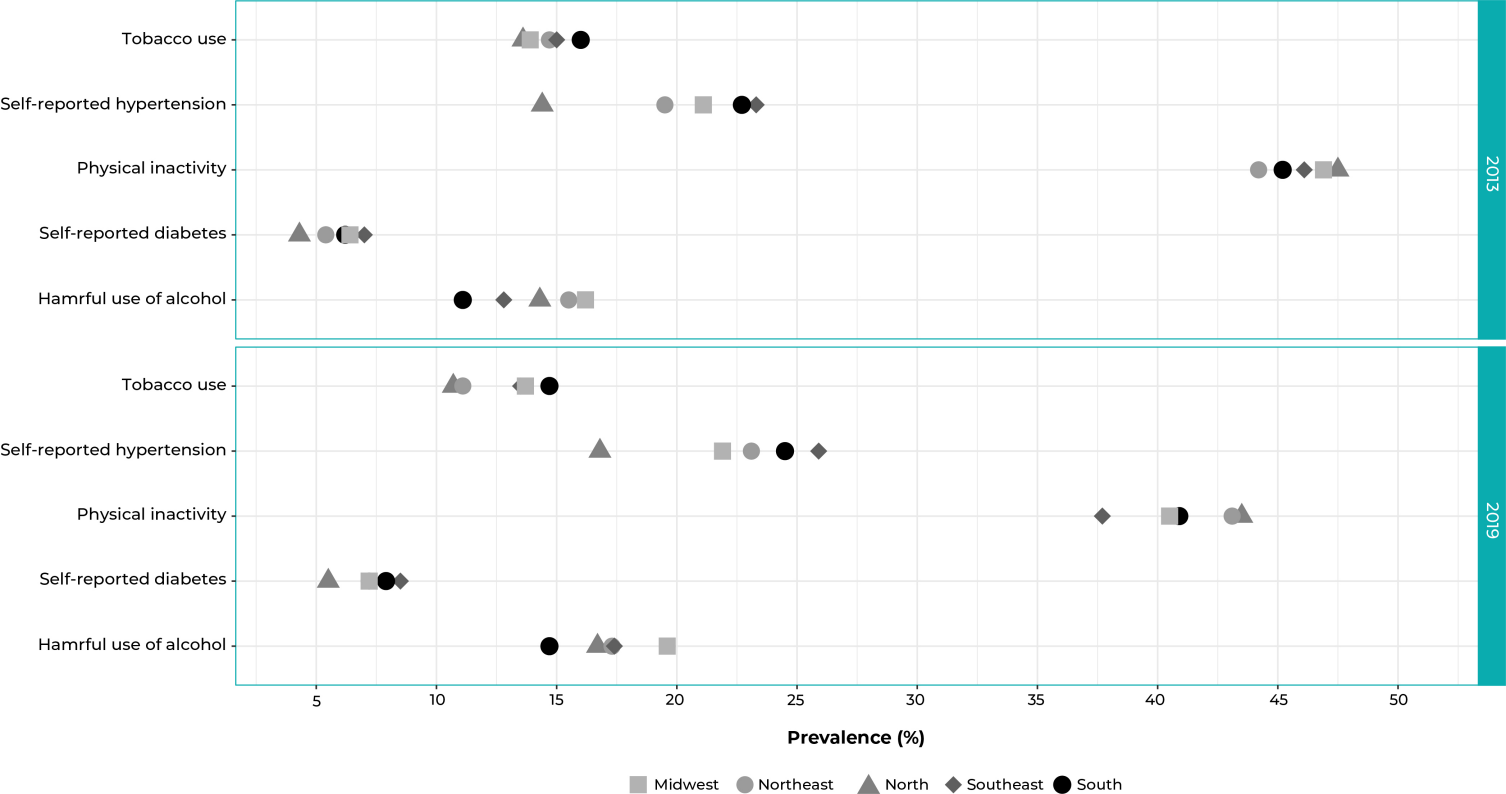
Prevalence of the indicators for monitoring the targets of the plans for tackling Non-Communicable Diseases (NCDs) in the Brazilian adult population, by geographic region, National Health Survey (PNS), 2013 (n = 60,202) and 2019 (n = 88,531)

When analyzing the targets according to the Brazilian regions, it is found that no region met the targets for alcohol abuse, high blood pressure, diabetes and tobacco use. Reducing physical inactivity by 10% was not achieved in the North and Northeast regions (Figure 2). Lastly, in terms of halting overweight and obesity, these targets were not measured considering the regions, only Brazil in its entirety.

## Discussion

There has been progress and some of WHO global plan targets may have been attained, namely, reduction of physical inactivity and increased cervical cancer screening (Pap smear) coverage. Conversely, the target for reducing the harmful use of alcohol was not attained, on the contrary, the prevalence increased. In terms of tobacco use, even though the prevalence declined, the target was not achieved. In addition, considering the data in this study, the target of halting the rise of high blood pressure, diabetes, overweight and obesity might not be attained by 2025.

Some indicators were only measured in 2013 (salt/sodium intake, measured blood pressure, measured diabetes by means of laboratory tests, drug therapy and cervical cancer screening coverage), thus, comparing the results is unfeasible. In the 2013 survey, sodium intake was shown to be high, therefore, its reduction will hardly be achieved. When stratifying by sex, education and region, inequalities were observed, thus, progressing equitably and providing improvements to all segments of the population turns out to be challenging. 

Alcohol use increased in both sexes for all levels of education and Brazilian regions. To try to reverse this scenario, some legislative measures were implemented in Brazil, such as the prohibition of the consumption of alcoholic beverages by automotive vehicle drivers, established through the “Lei Seca” (“Dry Law” or Prohibition Law) in 2008, and the New Prohibition Law in 2012, which increased the amount of the fine.^14^ Such measures resulted in decreased drinking and driving prevalence.^14^ However, there is still progress to be made in terms of the regulatory framework for alcohol in the country, especially in relation to the prohibition of beer advertising, considering that beer is not included in the marketing restriction because its alcohol content is below 13 degrees GL (Gay-Lussac).^14^

In the present study, even though a reduction in physical inactivity has been observed, its prevalence is still high, being one of the leading modifiable risk factors for NCDs, which negatively affects people’s mental health and life quality.^1^ ^,^ ^15^ In this respect, the Brazilian National Health Promotion Policy and the Strategic Action Plan to Tackle Non-communicable Diseases elected physical activity encouragement as its priority action and in 2011, the Health Gym Program (*Programa Academia da Saúde*) was criated,^16^ which allowed for an increase in physical activity and, consequentially, an improvement of the quality of life and health of the Brazilian population.^17^

Combating smoking has been considered successful, and Brazil has become an international reference in this respect. The advances have been attributed to the regulatory measures that have been adopted in the past years, such as the ban on smoking in public spaces, control of advertising and sponsorships, cigarette price increases, warning labels and images on cigarette packs, among others.^18^ In 1989, Brazil had a very high smoking prevalence (34.8%),^19^ in contrast, in the following years the tendency was a decline.^20^ In this regard, this study showed a prevalence of 12.8% in 2019. However, in order to achieve the 30% reduction target, in accordance with WHO Global Action Plan, restrictive regulatory measures must be accelerated.

The target of a 30% reduction in salt intake although not measured in 2019, is not expected to be reached. Important initiatives are highlighted, such as the launch of the Dietary Guidelines for the Brazilian Population (*Guia Alimentar para a População Brasileira*) in 2014^21^ and agreements to reduce sodium in processed foods.^22^ Nevertheless, if new regulatory measures are not effectively implemented, it is highly unlikely that the target will be attained. In this context, it is also worth highlighting the increase in obesity and, once again, the urgency of advancing in regulatory policies such as taxation of ultra-processed foods and regulation of food advertising to children, in addition to subsidies for the production of healthy products.^23^

The indicator related to drug therapy to prevent heart attacks and strokes, although not measured in 2019, had already been reached in 2013. Several initiatives implemented between 2011 and 2015 can explain this fact^6^ redefinition of the Care Network for People with NCDs (*Rede de Atenção às Pessoas com DCNTs*); the National Program for Improving Access and Quality of Primary Care (*Programa Nacional de Melhoria do Acesso e da Qualidade*); the Acute Myocardial Infarction Care (*Atenção ao Infarto Agudo do Miocárdio*); the Cerebrovascular Accident Care (*Atenção ao Acidente Vascular Cerebral*); the Popular Pharmacy Program; and the Better at Home Program (*Programa Melhor em Casa*), which aims to expand home care.^24^ These actions strengthened the Brazilian National Health System (SUS) response capacity and expanded actions aiming to prevent and control these NCDs. 

Strategies for tracking and early detection of cancer among women are beneficial and cervical cancer incidence, mortality and morbidity can be reduced by means of screening programs, health promotion actions, prevention, diagnosis and treatment.^25^ ^,^ ^26^ SUS ensures universal and free access to cervical cytology and Pap smears. This study showed that this target had also been attained in 2013.

Social inequalities regarding the RFs of NCDs can be the result of lack of opportunity in accessing health promotion and prevention practices, education and services.^27^ Populations with lower income and level of education, that live in poorer countries and regions, concentrate more NCDs and their RFs.^28^ The SDGs targets highlight the importance of intersectoral policies for the reduction of poverty, and also of gender and race/skin color differences, aiming for simultaneous progress of all countries. A pivotal milestone of the SDGs was “Leave no one behind”.^8^ In this context, this study shows inequalities and, in general, all the indicators were worse in the population with low schooling and residing in less socioeconomically developed regions. Therefore, it is crucial for Brazil to advance in a concerted way, reducing inequalities among its regions, as well as those related to gender, race/skin color and education. 

It is worth highlighting that the actions mentioned herein may have been discontinued due to Brazil’s economic and political crises (when austerity policies were implemented, such as the Constitutional Amendment No. 95 passed in 2016, freezing the budgets for social policies and health for 20 years, for instance), decrease of the gross domestic product and reduction of allocation of federal funds for health care to municipalities, which have thus affected the offer of health services.^25^ ^,^ ^26^ These factors disrupted social protection policies, contributed to an increase in poverty, extreme poverty and infant mortality, besides worsening health indicators.^25^ ^,^ ^26^ Austerity policies end up affecting the underprivileged and, as a consequence, intensify inequalities.^26^ In addition, there was no progress in terms of regulatory measures, which were abandoned after 2016. It should be noted that tobacco product prices have not increased in the past years and that surveillance activities have not been prioritized. Countries that have invested in regulatory measures have had significant progress, as is the case of Mexico, that taxed ultra-processed foods and, consequently, achieved a decline of 10% in the consumption of soft drinks.^29^

Among the limitations of this study, some indicators, such as measured blood pressure and biochemical measurements, were not collected in 2019, thus impairing the evaluation of such indicators in 2019, hence the use of self-reported data. Anthropometric measurements (weight and height), for the 2019 edition, were conducted in a subsample, allowing to evaluate overweight and obesity only at national level, not by region. Self-reported data can lead to under or overestimation of prevalences and generate less accurate estimates. Nevertheless, a study conducted with data from the city of Bambuí (state of Minas Gerais) cohort, aiming to determine the validity of self-reported diabetes and its determinants among elderly people, showed that self-reported data are reliable.^30^

There have been advances and improvements in the reduction of smoking and physical inactivity, in the provision of essential medications and coverage of Pap smears. However, some targets will not be met by 2025. Even though NCDs have gained priority in global and national agendas, challenges still remain for the development of effective policies for their control and prevention, combined with the need of advancing legal and regulatory measures. In the comprehensive approach to NCDs, intersectoral articulations and the reduction of socioeconomic and health inequalities are of the utmost importance.
